# There and back again, or always there? The evolution of rice combined strategy for Fe uptake

**DOI:** 10.3389/fpls.2014.00189

**Published:** 2014-05-14

**Authors:** Felipe K. Ricachenevsky, Raul A. Sperotto

**Affiliations:** ^1^Laboratório de Fisiologia Vegetal, Departamento de Botânica, Centro de Biotecnologia, Universidade Federal do Rio Grande do SulPorto Alegre, Brazil; ^2^Programa de Pós-Graduação em Biotecnologia, Centro de Ciências Biológicas e da Saúde, Centro Universitário UNIVATESLajeado, Brazil

**Keywords:** Fe uptake, combined strategy, molecular evolution, phylogenetic analyses, iron homeostasis

## Fe uptake mechanisms and transcriptional control

Iron (Fe) is an essential micronutrient for almost all living organisms and represents one of the most versatile metals in biology, being involved in many ubiquitous metabolic processes such as respiration and photosynthesis, and required as a co-factor for numerous enzymes (Sperotto et al., 2010; Grillet et al., 2014a). In plants, Fe deficiency can cause severe chlorosis, growth arrest, and even plant death. Although highly abundant in the earth's crust, Fe phytoavailability is usually low, mainly because Fe^3+^ forms insoluble Fe oxides (Lemanceau et al., 2009). To circumvent this problem, plants developed mechanisms to acquire Fe from the rhizosphere (Sperotto et al., 2012; Grillet et al., 2014a).

Fe uptake mechanisms were classically separate into two strategies. Strategy I, or reduction strategy, is carried out by all plants except those from Poaceae family, and consists of: (a) H^+^ extrusion by P-type ATPases to acidify the rhizosphere and increase Fe^3+^ solubility (e.g., AtAHA2, Arabidopsis H^+^-pump ATPase); (b) reduction of Fe^3+^ by a plasma membrane (PM)-bound ferric chelate reductase to more soluble Fe^2+^ (e.g., AtFRO2, Ferric Reductase Oxidase); and (c) Fe^2+^ absorption into root epidermal cells by transmembrane transporters (e.g., AtIRT1, Iron-Regulated Transporter) (Hindt and Guerinot, 2012; Ivanov et al., 2012). All three components of this strategy increase their activities during Fe deficiency. Strategy II, or chelation strategy, is used by plants from Poaceae family, and involves: (a) synthesis and release of small molecular weight compounds of the mugineic acid family called phytosiderophores (PS) into the rhizosphere, which bind Fe^3+^ with high affinity, via TOM1/OsZIFL4 (Nozoye et al., 2011; Ricachenevsky et al., 2011); and (b) Fe(III)-PS complex uptake into root cells by a Yellow Stripe/Yellow Stripe-Like (YSL) transporters. Both processes (PS excretion and Fe(III)-PS transport) are increased in response to Fe deficiency.

Since both maize (*Zea mays*) *ys1* mutant (defective for Fe(III)-PS transport) and *Arabidopsis thaliana irt1* mutant (defective for Fe^2+^ transport) cannot survive under Fe deficiency conditions, it was first widely accepted that these two Fe uptake strategies were the main mechanisms for Fe acquisition in each plant group. However, later work on rice (*Oryza sativa*) showed that two functional Fe^2+^ transporters, OsIRT1 and OsIRT2, were expressed in roots upon Fe deficiency (Ishimaru et al., 2006; Walker and Connolly, 2008). It was proposed that rice uses a combined strategy, which has all features of a strategy II plant (PS release through TOM1/OsZIFL4 and Fe(III)-PS uptake through OsYSL15, the YS1 ortholog—Inoue et al., 2009; Lee et al., 2009) and some features of a strategy I plant (Fe^2+^ uptake using IRT transporters). The other two components of strategy I plants, proton extrusion, and Fe(III)-chelate reductase activity, were not detected in Fe-deficient rice roots (Ishimaru et al., 2006). Further evidence for combined strategy was provided by rice plants carrying a mutation in the NICOTIANAMINE AMINOTRANSFERASE (NAAT) gene, a key enzyme in PS synthesis. This mutant, which lacks PS, is able to grow if Fe^2+^ is supplied as Fe source (Cheng et al., 2007). Based on these findings, it was proposed that the ability to absorb Fe^2+^ evolved in rice as an adaptation to the soil conditions in flooded paddies, where Fe^2+^ is more abundant than Fe^3+^ (Ishimaru et al., 2006; Walker and Connolly, 2008; Hindt and Guerinot, 2012). So far, rice is the only plant described to use the combined strategy mechanism.

A number of studies described key players and major transcriptional networks that control Fe homeostasis in both grasses and non-grasses (Hindt and Guerinot, 2012; Ivanov et al., 2012). Interestingly, orthologous genes have been described in rice and *Arabidopsis thaliana*, showing similar roles. The bHLH transcription factor FIT (FER-like iron-deficiency-induced transcription factor) from *A. thaliana* interacts with bHLH038 and bHLH039 to regulate IRT1 and FRO2 under Fe deficiency (Yuan et al., 2008). FIT has no ortholog in rice, but bHLH38/39 are highly similar to OsIRO2 (Hindt and Guerinot, 2012), a known downstream regulator of Fe deficiency-responsive genes. OsIRO2 regulates the Fe(III)-PS transport-related genes, but not OsIRT1 (Ogo et al., 2007). OsIDEF1, acting upstream of OsIRO2, and OsIDEF2, are transcriptional regulators of distinct but partially overlapping branches of Fe deficiency response in rice (Ogo et al., 2008; Kobayashi et al., 2009) However, no ortholog for OsIDEF1 or OsIDEF2 was described in *A. thaliana*, although similar genes are found in the genomic sequence (Kobayashi and Nishizawa, 2012).

In *A. thaliana*, a second regulatory network is controlled by bHLH transcription factor named POPEYE (PYE), which targets distinct metal homeostasis genes. PYE seems to be regulated by interacting partners such as BRUTUS (BTS), an E3 ubiquitin ligase with metal and DNA binding domains that negatively regulates the response to Fe deficiency (Long et al., 2010; Kobayashi et al., 2013). In rice, Zheng et al. (2011) identified a negative regulator of the Fe deficiency response, OsIRO3, which could be the ortholog of PYE (Hindt and Guerinot, 2012). Interestingly, BTS orthologs OsHRZ1 and OsHRZ2 were recently characterized as negative regulators of Fe uptake and Fe utilization genes (Kobayashi et al., 2013). Thus, it seems that control of Fe deficiency response is partly conserved between *A. thaliana* and rice. Moreover, Urzica et al. (2012) performed a trans-system analysis looking for genes responsive to low Fe supply in *Chlamydomonas reinhardtii*, *A. thaliana* and rice, and observed that BTS/HRZs, IRT1, and IRT2 are conserved throughout the plant lineage.

## Evolution of the Fe deficiency response

**Figure 1:**
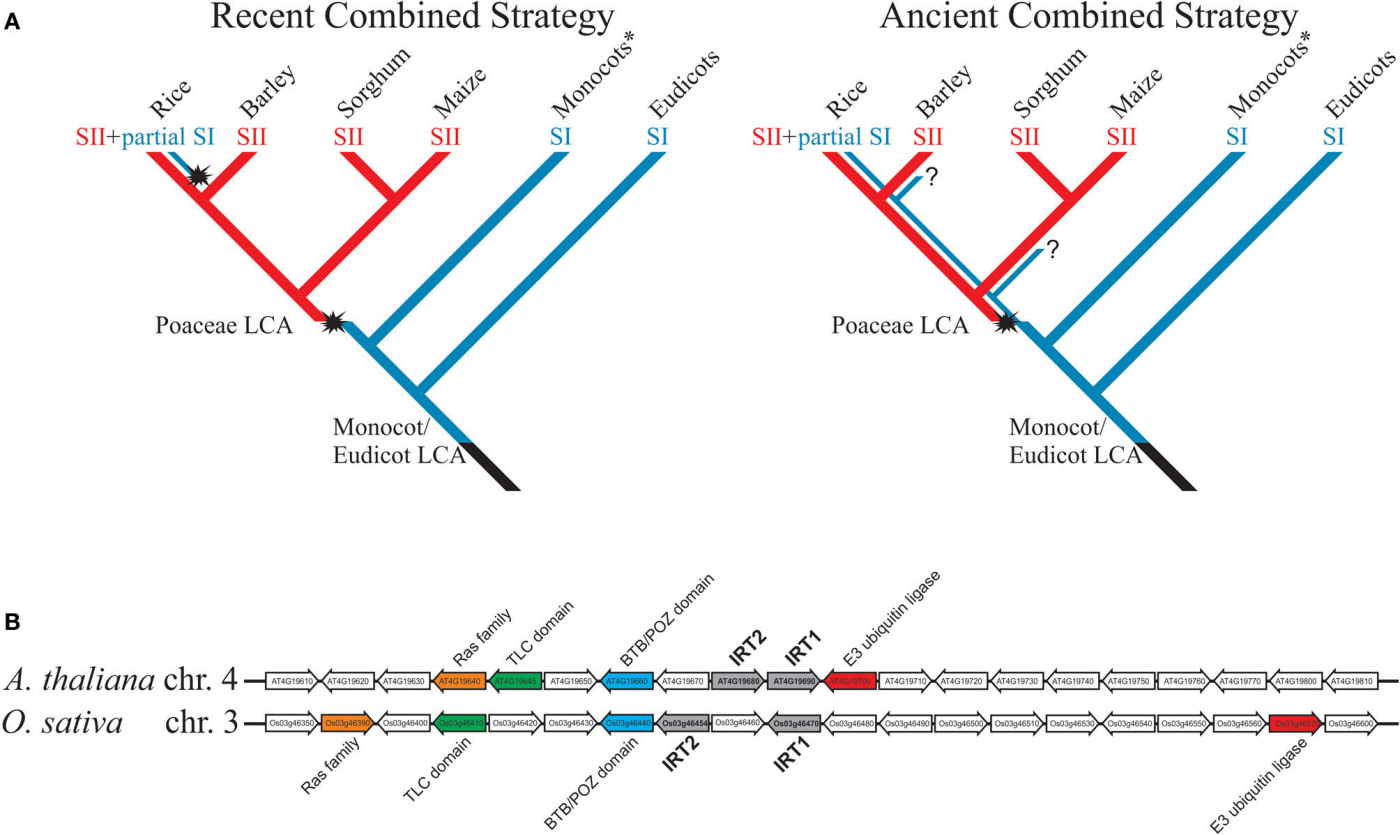
**(A)** Models for evolution of rice combined strategy for Fe uptake. Blue lines represent plants using strategy I for Fe uptake; red lines represent plants using strategy II. Evolutionary novelties are highlighted, as the appearance of strategy II in Poaceae group and the putative re-appearance of IRT1-mediated Fe^2+^ uptake in rice, also known as the combined strategy (strategy II + partial strategy I). Question marks denote unclear points. Monocots^*^ are all non-Poaceae monocotyledonous plants. SI = strategy I; SII = strategy II; LCA = last common ancestor. The selected monocot species are only to illustrate the divergence of Poaceae species, representing the subfamilies Ehrhartoideae (rice), Pooideae (barley), and Panicoideae (maize and sorghum). **(B)** Microsynteny between *AtIRT1*/*AtIRT2* and *OsIRT1*/*OsIRT2* loci. Conserved genes between rice and Arabidopsis were found using CoGe tool GEVo (http://genomevolution.org/CoGe/) and the Plant Genome Duplication Database (http://chibba.agtec.uga.edu/duplication/). Homologs are shown with same colors; *IRT1* and *IRT2* in each genome are in gray.

Currently there is no model for the evolution of Fe deficiency response in plants, especially in Poaceae, and few studies have focused on testing the hypothesis that rice is the only combined strategy species. Considering the available evidence, two models are possible (Figure 1A). In the first, named “recent combined strategy,” combined strategy is an evolutionary novelty restricted to rice or close ancestral species, not shared with other extant species from Poaceae, which all use strategy II, and rice has acquired ability to induce an IRT1-like transporter under Fe deficiency, partially resembling strategy I, as an adaptation to flooding. It implies that Poaceae last common ancestor (LCA) has lost strategy I-based Fe acquisition capacity and gained strategy II-based Fe uptake mechanism before diversification within the family (Figure 1A).

In the second model, named “ancient combined strategy,” combined strategy is considered an evolutionary ancient trait (Figure 1A). Poaceae LCA has gained the ability to use strategy II for Fe uptake, but maintained strategy I. During Poaceae diversification and speciation, both strategy I and strategy II-specific genes were available for natural selection, and thus distinct groups could have adapted differently to respond to low Fe condition. As an example, the rice lineage has maintained IRT1-like up-regulation from the original strategy I response, while rhizosphere acidification and Fe reduction traits were lost. While all Poaceae would use the more efficient Fe acquisition mechanism strategy II (Curie and Briat, 2003), the model predicts that extant species could also show partial strategy I as rice does (i.e., IRT1-like Fe^2+^ uptake).

Indirect evidence favors the ancient combined strategy model. *OsIRT1* ortholog in barley (*Hordeum vulgare*), *HvIRT1*, is also up-regulated by Fe deficiency and transports Fe, Mn, Zn, and Cd (Pedas et al., 2008). The maize ortholog, *ZmIRT1*, was both described as not Fe regulated by Nozoye et al. (2013) and as Fe regulated by Li et al. (2013). Either way, Li et al. (2013) observed strong up-regulation of ZmIRT1 under Fe deficiency and showed ZmIRT1 ability to complement Fe and Zn uptake-defective yeast strains. Interestingly, *IRT1*-like genes described in Poaceae clustered together with *AtIRT1* in a phylogenetic analysis (Li et al., 2013). *AtIRT2* and *OsIRT2*, two genes similar to *AtIRT1* and *OsIRT1* that code for Fe transporters (Ishimaru et al., 2006; Vert et al., 2009), are part of the same cluster. Strikingly, *AtIRT1*/*AtIRT2* and *OsIRT1*/*OsIRT2* gene pairs are localized in tandem in their respective genomic regions and, despite monocot/dicot divergence dates 120 to 200 million years ago (Salse et al., 2002), they still show some degree of microsynteny (Figure 1B). These data indicate that OsIRT1 (and probably OsIRT2) shares not only functional similarity but also common evolutionary origin with AtIRT1, as the ancient combined strategy model predicts. Moreover, phenolics were described as important for Fe deficiency response in *A. thaliana* and rice, indicating that less understood aspects of Fe deficiency response are conserved between strategy I and strategy II plants (Bashir et al., 2011; Rodríguez-Celma et al., 2013; Fourcroy et al., 2014; Schmid et al., 2014).

We should also consider the likeliness of each model. In recent combined strategy model, the LCA *IRT1* gene would have lost Fe deficiency responsiveness, presumably through deleterious mutations related to promoter activity (i.e., hampering transcription factor binding, interaction with enhancers, etc.). For *OsIRT1* to be able to respond again to low Fe concentration in combined strategy, such mutations (or any changes that rendered IRT1 non-regulated) would need to be reversed. That implies re-activation of non-functional regulatory sequences on the promoter of the same gene, which is part of the *ZIP* gene family of around ten members in Poaceae genomes, several of them encoding Fe transporters (Li et al., 2013), and re-insertion into intricate regulatory circuits (Kobayashi and Nishizawa, 2012). Although possible, the ancient combined strategy model is more parsimonious, predicting that IRT1 function was conserved through plant lineage evolution, and that preference for combined strategy or strategy II in Poaceae was a late adaptation (Figure 1A).

## Conclusions and future perspectives

It has long been established that Poaceae species rely on strategy II mechanism, while all other plant groups use strategy I. Rice has been considered an exception to the Poaceae-strategy II rule (Ishimaru et al., 2006; Sperotto et al., 2012); however, although it is clear that strategy II is the main mechanism for Fe uptake in graminaceous plants, the presence of IRT1-based Fe^2+^ transport in roots might play a non-overlapping role, not only in rice but in other species (Ishimaru et al., 2006; Pedas et al., 2008; Li et al., 2013). The ancient combined strategy model proposed here states that AtIRT1 ortolog kept their ancient function, observed early in the plant lineage (Urzica et al., 2012), all the way from monocot/eudicot split to extant *Oryza sativa* species, rather than re-emerged with the same function in rice (Figure 1). Then, it is possible that distinct Poaceae subfamilies and species evolved independently to strategy II-exclusive or combined strategy-like strategies.

Many studies have focused on Fe acquisition genes and underlying signaling pathway controlling these strategies, but most available data is on model species *A. thaliana* and rice (Hindt and Guerinot, 2012; Ivanov et al., 2012; Kobayashi and Nishizawa, 2012). Besides these, studies in maize, barley and *Brachypodium distachyon* have indicated the role of YSL transporters in Fe(III)-PS acquisition (Curie et al., 2001; Murata et al., 2006; Yordem et al., 2011). However, the role of IRT1-like transporters is still poorly understood. Studies on the role of IRT1, as well as other strategy I-related genes such as Fe^3+^-reductase, in Poaceae species, should shed light into how exclusive rice combined strategy is.

We should also consider that other species might use variants of strategy I and strategy II, or even rely on distinct mechanisms. Recently, it was demonstrated that peanut (*Arachis hypogaea*), an eudicot, is able to absorb Fe(III)-PS complexes through AhYSL1 transporter. The complexes, however, are only present after intercropping with maize, which secretes PS in the soil, increasing Fe efficiency of peanut (Xiong et al., 2013). An exciting new study described a previously unknown Fe uptake mechanism, where Fe is delivered to embryos of pea (*Pisum sativum*) and *A. thaliana* in the form of Fe(III)-malate/citrate complexes, and is then chemically reduced to Fe^2+^ by ascorbate, which is effluxed from embryos, for subsequent uptake (Grillet et al., 2014b).

With lowering costs and increased access to technologies such as next-generation sequencing (Mardis, 2013) and genome editing (Gaj et al., 2013), it is becoming feasible to perform comparative genomics and transcriptomic studies with plants species for which genetic resources are not available, and eventually test key genes identified. These comparisons will allow testing the models discussed here, as well as uncovering new genes and strategies for Fe acquisition.

### Conflict of interest statement

The authors declare that the research was conducted in the absence of any commercial or financial relationships that could be construed as a potential conflict of interest.
